# Time from admission to initiation of surgery for source control is a critical determinant of survival in patients with gastrointestinal perforation with associated septic shock

**DOI:** 10.1186/cc13854

**Published:** 2014-05-02

**Authors:** Takeo Azuhata, Kosaku Kinoshita, Daisuke Kawano, Tomonori Komatsu, Atsushi Sakurai, Yasutaka Chiba, Katsuhisa Tanjho

**Affiliations:** 1 Department of Acute Medicine, Division of Emergency and Critical Care Medicine, Nihon University School of Medicine, 30-1 Oyaguchi Kamimachi Itabashi-ku, Tokyo 173-8610, Japan; 2 Division of Biostatistics, Clinical Research Center, Kinki University School of Medicine, 377-2 Onohigashi, Sayama-shi, Osaka 589-8511, Japan

## Abstract

**Introduction:**

We developed a protocol to initiate surgical source control immediately after admission (early source control) and perform initial resuscitation using early goal-directed therapy (EGDT) for gastrointestinal (GI) perforation with associated septic shock. This study evaluated the relationship between the time from admission to initiation of surgery and the outcome of the protocol.

**Methods:**

This examination is a prospective observational study and involved 154 patients of GI perforation with associated septic shock. We statistically analyzed the relationship between time to initiation of surgery and 60-day outcome, examined the change in 60-day outcome associated with each 2 hour delay in surgery initiation and determined a target time for 60-day survival.

**Results:**

Logistic regression analysis demonstrated that time to initiation of surgery (hours) was significantly associated with 60-day outcome (Odds ratio (OR), 0.31; 95% Confidence intervals (CI)), 0.19-0.45; *P* <0.0001). Time to initiation of surgery (hours) was selected as an independent factor for 60-day outcome in multiple logistic regression analysis (OR), 0.29; 95% CI, 0.16-0.47; *P* <0.0001). The survival rate fell as surgery initiation was delayed and was 0% for times greater than 6 hours.

**Conclusions:**

For patients of GI perforation with associated septic shock, time from admission to initiation of surgery for source control is a critical determinant, under the condition of being supported by hemodynamic stabilization. The target time for a favorable outcome may be within 6 hours from admission. We should not delay in initiating EGDT-assisted surgery if patients are complicated with septic shock.

## Introduction

It is difficult to determine the best time to initiate surgery for GI perforation with associated septic shock. It is common to stabilize circulatory dynamics before surgery [1]; however taking a long time to initiate surgery may result in death from sepsis [2]. Even if circulatory dynamics are not stabilized, should we initiate surgery early? No clinical data indicating an answer have been published. Early goal-directed therapy (EGDT) is a procedure for the initial resuscitation of patients with septic shock that specifies targets to be reached within 6 hours of admission [3]. It is appreciated that EGDT can speedily improve the circulatory dynamics and in-hospital mortality of patients with septic shock [3-5]. However, EGDT patients who required immediate surgery, such as septic shock patients with gastrointestinal (GI) perforation and diffuse peritonitis, were excluded in the original article [3]. This may have been because the adequacy of the surgical intervention may have had a significant influence on the outcomes of these patients [6].

We hypothesized that the outcomes of patients with GI perforation with associated septic shock could be improved by initiating surgery immediately after admission in order to control the infectious lesions entirely (early source control) with the support of early hemodynamic stabilization by initial resuscitation in accordance with EGDT. Therefore, we developed a protocol including early source control and EGDT for GI perforation with septic shock.

The primary goal of this study was to statistically demonstrate the relationship between time from admission to initiation of surgery and 60-day outcome. The secondary goal was to determine a target time from admission to initiation of surgical intervention for a favorable outcome in patients with GI perforation with associated septic shock.

## Methods

### Study design and setting

A prospective observational study was conducted in the emergency department (ED), operating room (OR), and ICU of the emergency and critical care center (E&CCC) of an urban academic medical center, a 1000-bed teaching hospital with more than 100,000 patients visits per year. The E&CCC is specifically designed for patients with life-threatening conditions (shock, severe trauma, cardiopulmonary arrest, acute myocardial infarction, et cetera) who are delivered by ambulance, and is equipped for initial treatment, diagnostic imaging, surgery, and intensive care. Approximately 2,200 to 2,400 patients per year are delivered to the E&CCC, including approximately 100 with severe acute abdominal conditions. Approximately 50 abdominal surgical operations per year are performed to treat GI perforation and abdominal trauma by three surgeons who belong exclusively to the E&CCC and who are certified by both the Japanese Society of Surgery and the Japanese Society of Emergency Medicine.

**Figure 1:**
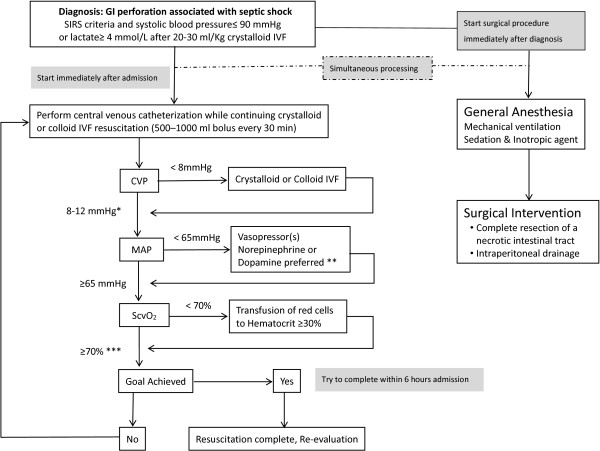
**Protocol for gastrointestinal perforation with associated septic shock.** The protocol for early infectious source control (EISC) and early goal-directed therapy (EGDT) for gastrointestinal perforation with septic shock was implemented at Nihon University Itabashi Hospital. GI, gastrointestinal; SIRS: systemic inflammatory response syndrome; IVF, intravenous fluids; CVP, central venous pressure; MAP, mean arterial pressure; ScvO_2_, central venous oxygen saturation. Revised points from the original protocol of Rivers *et al*. [3]; *in mechanical ventilation control, the target CVP is ≥8 mm Hg; **the original protocol specified dobutamine, but this was not used; ***blood gas analysis (BGA) measurement of ScvO_2_ in blood drawn from the internal jugular vein via an indwelling catheter.

The aim of this protocol is to initiate complete infectious source control immediately after admission of patients with GI perforation and associated septic shock, concomitant with hemodynamic management. We define this intervention as early source control. Early improvement of hemodynamics is required for early source control and initial resuscitation performed in accordance with EGDT, which was described by Rivers *et al*. [3] and aims for central venous pressure (CVP) of 8 to 12 mm Hg, mean arterial pressure (MAP) ≥65 mm Hg, and central venous oxygen saturation (ScvO_2_) ≥70% within 6 hours of admission. Therefore, our protocol specifies that surgery should start immediately with the performance of EGDT. The EGDT method was partially revised from the original procedure to allow its performance simultaneously with early source control. The first revision was to the method of determining ScvO_2_. Due to difficulties in continuous monitoring simultaneous with surgery, blood gas analysis (BGA) was conducted using blood drawn from the internal jugular vein via a central venous catheter every 30 minutes (at admission and from initiation to completion of surgery). The second revision was associated with target setting for CVP under mechanical ventilation. In our protocol we specify a level of ≥8 mm Hg, as our patients are not under mechanical ventilation to control respiration but to provide general anesthesia (Figure 1).

The patients in this examination were transferred to the E&CCC between January 2007 and December 2011 and treated in accordance with the protocol. The details of the therapeutic protocol, including surgery, were shown to all of the patients or their families before the surgery and informed consent was obtained. The purpose and method of this examination were explained and we obtained the informed consent for all patients. This examination was reviewed and approved by the Research review board of Nihon University School of Medicine, Itabashi Hospital.

### Patient registration

This study was intended for patients with GI perforation associated with septic shock, whose infectious sources were controlled by surgical procedures completely assisted by EGDT resuscitation. Accordingly, the registration criteria for enrollment were: 1) age ≥18 years old with GI perforation (stomach, duodenum, small intestine, colon, or rectum); 2) complicated by shock; 3) initial resuscitation using EGDT performed in the ED according to the protocol; 4) complete resection of a necrotic intestinal tract and irrigation/drainage for peritonitis; and 5) postoperative intensive care in the ICU. The definition of shock was in accordance with that of Rivers *et al*. [3]: fulfillment of two of four criteria for systemic inflammatory response syndrome (SIRS) and systolic blood pressure no higher than 90 mm Hg (after a crystalloid-fluid challenge of 20 to 30 ml per kg of body weight over a 30-minute period) or a blood lactate concentration of ≥4 mmol/L (Figure 1).

### Treatment

For diagnosis of GI perforation and intestinal necrosis, contrast-enhanced multi-slice helical computed tomography (CT) was performed in all patients. A radiographic contrast study with aqueous contrast medium was added on the judgment of the physician. Surgery was started for all patients as soon as the patient was diagnosed, regardless of circulatory dynamics. All surgical procedures were performed by the surgeons of the E&CCC. All of the surgery was performed using a single methodology, in accordance with our protocol, by either the study director or one two surgeons working under the supervision of the study director. All three of these surgeons cooperated with this examination, fully understood its purposes and methods, and practiced exclusively at the E&CCC. For patients with upper GI perforation, omental plication for perforated gastric or duodenal peptic ulcer and abdominal irrigation/drainage were performed. When gastric perforation was caused by cancer, omental plication and a secondary radical operation were performed. For all patients with lower digestive perforations, complete resection of the necrotic intestinal tract and irrigation/drainage for peritonitis with no transient primary anastomosis were performed. Even if the necrotic segment of the intestine was small, for example, perforation with colonic diverticulitis or a small intestinal ulcer, we performed a partial intestinal resection. The extent of peritonitis was evaluated by identifying the area in which turbid ascites contaminated with intestinal juice or stool was present. For patients who had bowel dilation, we inserted an ileus tube during laparotomy to perform intestinal depression. We obtained two sets of blood cultures prior to administration of antibiotics and two sets of ascites cultures during surgery. In addition to standard cultures, we used the 1, 3 β-D-glucan assay to diagnose systemic fungal infection. Broad-spectrum antibiotics such as carbapenem (meropenem hydrate (MEPM), doripenem hydrate (DRPM)) and tazobactam/piperacillin (TAZ/PIPC) were administered to all patients immediately after diagnosis and during surgery. As additional options, vancomycin (VCM) and antifungal agents were used perioperatively when the patients were suffering mainly from healthcare-associated intra-abdominal infection, and who were known to be colonized with the organism or who at risk of having an infection due to this organism because of prior treatment failure and significant antibiotic exposure. We defined the initiation of antimicrobial therapy as the administration of the first antimicrobial agent. We considered the appropriateness of initial antimicrobial therapy with reference to the identified causative pathogen and susceptibility testing. A central venous catheter was inserted into the internal jugular vein on admission to the ER. To monitor arterial blood pressure continuously and to conduct BGA, including blood lactate concentration in arterial blood, a catheter was inserted into the radial artery on admission. The initial infusion was given in accordance with EGDT [3]. A 500- to 1,000-ml bolus of crystalloid fluid was given every 30 minutes to achieve a CVP of 8 to 12 mm Hg. Sedation was performed simultaneously with mechanical ventilation. If the MAP was <65 mmHg, vasopressors were given to maintain a MAP ≥65 mm Hg. Norepinephrine and dopamine were used as vasopressors. If ScvO_2_ was <70%, red blood cells were transfused to achieve a hematocrit (Hct) level ≥30%.

### Measures

Body temperature, pulse rate, blood pressure, and urine output were determined at hourly intervals. Biochemical and coagulation tests were conducted on admission, immediately after surgery and on the morning after surgery, and BGA, including lactate, was conducted at intervals of one hour. These data were used to calculate the acute physiology and chronic health evaluation II (APACHE II) score [7] as an indicator of severity, and the sequential organ failure assessment (SOFA) score [8,9] and the multiple organ dysfunction (MOD) score [10] as indicators of organ failure. In calculating these scores, the worst test data within 24 hours after admission were used. The blood lactate concentration reflects the severity of sepsis and is correlated with outcome [11]; therefore, this value is commonly used as an indicator for the treatment of sepsis [12,13]. ScvO_2_ is an indicator of tissue oxygenation [14] and reflects septic conditions well [15,16]. Furthermore, it has been demonstrated that a low level of initial ScvO_2_ is associated with high mortality in septic patients [17]. Patients were followed up for 60 days or until death. Patients who were discharged within 60 days were followed up by telephone.

### Statistical analysis

Logistic regression analysis was used to examine 60-day survival as a function of time from admission to initiation of surgery using interval data. We calculated the *P*-value, odds ratio and 95% CI. *P* <0.05 was considered significant.

We performed multivariate logistic regression analysis to reduce the influence of potential confounding factors. Variables with *P*-values <0.2 on bivariate analysis were then introduced into the multivariate model [18]. All patients were classified as survivors or non-survivors on day 60. Patient background (age, gender), perforation site, severity on admission (SOFA score, APACHE II score, MOD score, MAP on admission, blood lactate on admission, and ScvO_2_ on admission), extent of peritonitis, fluid resuscitation (infusion volume until 2 hours and 6 hours after admission), antimicrobial therapy (appropriateness of initial antimicrobial therapy and time from admission to initiation of antimicrobial therapy), and surgical factors (time from admission to initiation of surgery, duration of surgery and re-laparotomy) were compared by bivariate analysis. Re-laparotomy includes both planned laparotomy and unplanned laparotomy with the exception of the secondary radical operation for gastric cancer. The Fisher or Pearson exact test was performed for categorical variables and the unpaired *t*-test was performed for continuous variables. Continuous data are presented as means ± SD. Multi-colinearity, assessed using variance inflation factors [19], was detected between age and APACHE II score, and among APACHE II score, SOFA score and MOD score; these variables were not included separately in the multivariate model. Multiple logistic regression analysis yielded the *P*-value, odds ratio and 95% CI. We considered *P*-values <0.05 as significant differences in the multivariate model. Statistical analysis was performed using JMP ver. 9.0.3 (SAS Institute, Cary, NC, USA).

We classified all patients into 2-hour groups (from 0 to 12 hours) from admission to initiation of surgery and calculated the number of survivors and non-survivors and the survival rate at 60 days for each group. Furthermore, we determined the target time from admission to initiation of surgery, which was associated with a favorable 60-day outcome.

## Results

Over the observation period from 2007 to 2011, a total of 154 patients met the registration criteria and were enrolled in the study. All of the surgery was performed using a single methodology. The primary diseases of all patients were assessed (Table 1). The major causes of GI perforation were colon/rectal diverticulitis, mechanical small bowel obstruction and mesenteric ischemia. The mortality in mesenteric ischemia was characteristically high at 26.5%. The baseline characteristics and outcomes of all patients were evaluated (Table 2). The age of the patients was 66.5 ± 13.9 years, and 57.1% of the patients were men. The major sites of perforation were the small intestine (42.9%) and colon (40.9%). Meanwhile, there were a few patients (9.1% in total) with upper GI tract (stomach and duodenum) perforation. We found that all severity scores (SOFA score, APACHE II score and MOD score) were high and that the patients presented with low MAP (66.2 ± 29.9 mm Hg) and high blood lactate concentration (5.69 ± 4.03 mmol/L). We isolated representative Gram-positive, Gram-negative and anaerobic bacterium and yeast/fungi in blood and ascites cultures. We confirmed that there were 20 patients with *Pseudomonas aeruginosa* (13.0%), 11 patients with methicillin-resistant *Staphylococcus aureus (MRSA)* (7.1%), eight patients with *Enterococcus spp.* (5.2%) and 11 patients with yeast/fungi (7.1%). Many patients had mixed infections. By bacteriological examination, we judged that 124 patients (80.5%) received appropriate initial antimicrobial therapy. We confirmed that the surgery was performed using a single methodology according to the protocol, that 3.1 ± 1.5 hours were needed up to the initiation of the surgery, and that 3.4 ± 1.4 hours were required for the surgery itself. Patients with peritonitis in three or four quadrants represented more than 95.5% of the total. Conversely, no patients had abscesses or peritonitis in one quadrant. No patient underwent damage control surgery and two patients underwent an open abdominal technique: 18 patients (11.7%) required additional re-laparotomy. Finally, the survival ratio was 82.5% on day 28 and 77.9% on day 60.

**Table 1:** Primary diseases of all patients

	**Patients, number (%)**	**Deaths, number (%)**
Colon/rectal diverticulitis	35 (22.7)	6 (17.6)
Mechanical small bowel obstruction	27 (17.5)	3 (8.8)
Mesenteric ischemia and necrotic bowel	21 (l3.6)	9 (26.5)
Idiopathic lower digestive tract perforation	16 (10.4)	5 (14.7)
Colon/rectal cancer	15 (9.7)	0 (0.0)
Gastric/duodenal peptic ulcer	9 (5,8)	1 (2.9)
Non-occlusive mesenteric ischemia	9 (5.8)	4 (11,8)
Gastric canes	5 (3.2)	1 (2.9)
Inflammatory bowel disease	5 (3.2)	1 (2.9)
Sigmoid volvulus	3 (1.9)	0 (0.0)
Strangulated inguinal/femur hernia	3 (1.9)	0 (0.0)
Toxic mega-colon	2 (1.3)	2 (5.9)
Other	4 (2.6)	2 (5.9)
Total	154	34

**Table 2:** Baseline characteristics of all patients

**Number of patients: 154**	
**• Patient background**	
Age, years, mean ± SD	66.5 ± 13.9
Male:female ratio	88:66
**• Perforation site, number of patients (%)**	
Small intestine	66 (42.9)
Colon	63 (40.9)
Stomach	8 (5.2)
Duodenum	6 (3.9)
Rectum	7 (4.5)
Combinations	4 (2.6)
**• Severity on admission, mean ± SD**	
SOFA score	9.14 ± 3.78
APACHE-II score	24.0 ± 8.62
MOD score	4.77 ± 3.23
MAP on admission, mmHg	66.2 ± 29.9
Blood lactate concentration on admission, mmol/L	5.69 ± 4.03
ScvO_2_ on admission, %	58.9 ± 12.4
**• Extent of peritonitis, number of patients (%)**	
Abscess	0 (0)
One quadrant	0 (0)
Two quadrants	9 (5.8)
Three quadrants	76 (49.4)
Four quadrants	71 (44.8)
**• Fluid resuscitation, mean ± SD**	
Infusion volume within 2 hours of admission, ml	2858.8 ± 587.5
Infusion volume within 6 hours of admission, ml	5125.2 ± 712.1
**• Microbiology, number of patients (%)**	
*Escherichia coli*	75 (48.7)
*Proteus spp.*	20 (13.0)
*Klebsiella spp.*	16 (10.4)
*Pseudomonas aeruginosa*	20 (13.0)
Methicillin-resistant *Staphylococcus aureus (MRSA)*	11 (7.1)
*Enterococcus spp.*	8 (5.2)
*Bacteroides spp.*	42 (27.3)
*Clostridium spp.*	6 (4.0)
Yeast/fungi	11 (7.1)
**• Antimicrobial therapy**	
Initial antimicrobial therapy, appropriate:inappropriate ratio	124:30
Time from admission to initiation of antimicrobial therapy, hours*, mean ± SD	2.3 ± 0.3
**• Surgery**	
Time from admission to initiation of surgery, hours, mean ± SD	3.1 ± 1.5
Duration of surgery, hours, mean ± SD	3.4 ± 1.4
Addition of re-laparotomy**, number of patients (%)	18 (11.7)
Damage control laparotomy, number of patients (%)	0 (0)
Open abdominal technique, number of patients (%)	2 (1.3)
**• ICU stay, days, mean ± SD**	34.7 ± 36.9
**• Surviving patients, number (%)**	
28-day	127 (82.5)
60-day	120 (77.9)

SOFA, sequential organ failure assessment; APACHE-II, acute physiology and chronic health evaluation II; MOD, multiple organ dysfunction; MAP, mean arterial pressure; ScvO_2_, central venous oxygen saturation. ^*^Initiation of antimicrobial therapy was defined as administration of the first antimicrobial agent to the patient. ^**^Re-laparotomy includes both planned laparotomy and unplanned laparotomy with the exception of secondary radical operation for gastric cancer.

In logistic regression analysis between time from admission to initiation of surgery and 60-day outcome, time to initiation of surgery was significantly associated with 60-day outcome (adjusted odds ratio 0.31 (per hour delay), 95% CI 0.19, 0.45; *P*-value <0.0001).

The patients were classified as survivors or non-survivors and characteristics of the patients were compared by bivariate analysis (Table 3). Between the two groups there were significant differences (*P*-value <0.2) in the bivariate model in age, severity on admission (SOFA score, APACHE-II score, MOD score, blood lactate concentration and ScvO_2_), time to initiation of surgery, and infusion volume over a 2-hour period. Furthermore, all variables with significant differences were taken forward to multiple logistic regression analysis. In accordance with the statistical method of this examination, we chose the SOFA score, which had the lowest *P*-value among the SOFA, APACHE-II and MOD scores. We identified two independent factors associated with 60-day survival: SOFA score (adjusted odds ratio 0.80, 95% CI, 0.66, 0.95; *P* = 0.014) and time from admission to initiation of surgery (adjusted odds ratio 0.29 (per hour delay), 95% CI 0.16, 0.47; *P* <0.0001).

**Table 3:** Comparison of survivors and non-survivors

	**Bivariate model**		**Multivariate model**			
	**Survivors (n = 120)**	**Non-survivors (n = 34)**	***P*-value**	**Odds ratio**	**95% CI**	***P*-value**
**• Patient background**						
Age, years, mean ± SD	64.3 ± 14.4	74.1 ± 8.7	0.0002*	0.95	0.88, 1.00	0.071
Male:female ratio	68:52	20:14	0.82			
**• Perforation site, number of patients (%)**						
Small intestine	51 (42.5)	15 (44.1)	0.76			
Colon	49 (40.8)	14 (41.2)				
Stomach	7 (5.8)	1 (2.9)				
Duodenum	5 (4.2)	1 (2.9)				
Rectum	6 (5.0)	1 (2.9)				
Combinations	2 (1.7)	2 (5.9)				
**• Severity on admission, mean ± SD**						
SOFA score	8.5 ± 3.3	11.5 ± 4.3	<0.0001*	0.80	0.66, 0.95	0.014**
APACHE-II score	22.8 ± 8.5	28.1 ± 7.9	0.0014*	-	-	-
MOD score	4.3 ± 2.9	6.5 ± 3.7	0.0004*	-	-	-
Mean arterial pressure on admission, mm Hg	62.3 ± 29.5	62.2 ± 31.6	0.38			
Blood lactate concentration on admission, mmol/L	5.0 ± 3.6	8.1 ± 4.4	<0.0001*	0.88	0.77, 1.01	0.078
ScvO_2_ on admission, %	60.8 ± 11.8	52.2 ± 12.1	0.0003*	1.04	0.99, 1.09	0.13
**• Extent of peritonitis, number of patients (%)**						
Abscess	0 (0)	0 (0)	0.95			
One quadrant	0 (0)	0 (0)				
Two quadrants	7 (5.8)	2 (5.9)				
Three quadrants	60 (50.0)	16 (47.1)				
Four quadrants	53 (44.2)	16 (47.1)				
**• Fluid resuscitation, mean ± SD**						
Infusion volume within 2 hours of admission, ml	2915.8 ± 618.5	2657.4 ± 409.0	0.023*	1.00	1.00, 1.00	0.17
Infusion volume within 6 hours of admission,ml	5153.8 ± 694.0	5024.3 ± 775.2	0.35			
**• Antimicrobial therapy**						
Patients with inappropriate initial antimicrobial therapy, number (%)	23 (19.2)	7 (20.6)	0.85			
Time from admission to initiation of antimicrobial therapy, hours^#^	2.3 ± 0.32	2.3 ± 0.38	0.48			
**• Surgery**						
Time from admission to initiation of surgery, hours, mean ± SD	2.6 ± 1.0	4.6 ± 1.6	<0.0001*	0.29	0.16 – 0.47	<0.0001**
Duration of surgery, hours, mean ± SD	3.4 ± 1.4	3.4 ± 1.4	0.92			
Patients needing re-laparotomy^##^, number (%)	13 (10.8)	5 (14.7)	0.54			
Patients needing open abdominal technique, number (%)	1 (0.8)	1 (2.9)	0.33			
		*P-value <0.2				**P-value <0.05

All variables with *P*-value <0.2 in the bivariate model were taken forward to the multivariate model (multiple logistic regression analysis). * meant p-value was less than 0.2. The SOFA score was chosen for the multivariate model from among the SOFA, APACHE-II and MOD scores which had multi-colinearity. The SOFA score and time from admission to initiation of surgery were selected as independent factors associated with 60-day survival by multiple logistic regression analysis with a *P*-value <0.05. ** meant p-value was less than 0.05. SOFA, sequential organ failure assessment; APACHE-II, acute physiology and chronic health evaluation II; MOD, multiple organ dysfunction; MAP, mean arterial pressure; ScvO_2_, central venous oxygen saturation. ^#^Initiation of antimicrobial therapy was defined as administration of the first antimicrobial agent to the patient. ^##^Re-laparotomy includes both planned laparotomy and unplanned laparotomy with the exception of secondary radical operation for gastric cancer.

**Figure 2:**
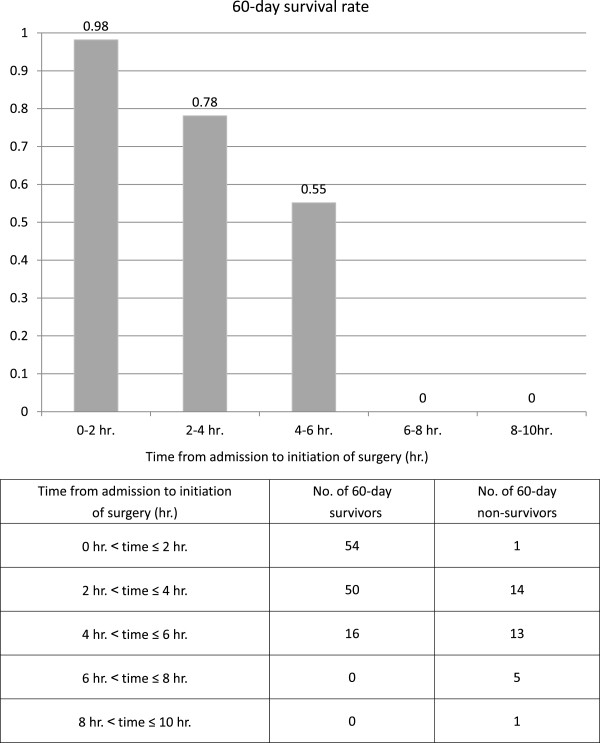
**Time from admission to initiation of surgery and 60-day outcome.** All patients were classified into 2-hour groups (from 0 to 12 hours) from admission to initiation of surgery. The number of survivors and non-survivors and the survival rate on day 60 are shown. As the time to initiation of surgery increased, survival rate decreased and the survival rate was 0% in the group that waited more than 6 hours. There were no patients who needed more than 10 hours to initiate surgery.

All 154 patients indicated for the protocol were classified according to time from admission to initiation of surgery, which we divided into 2-hour periods, and we compared the number of survivors, the number of non-survivors, and the survival rate for each at 60 days (Figure 2). Of the 55 patients in which surgery was started within the first 2 hours, the 60-day survival rate was 98%. As the time to initiation of surgery increased, the survival rate decreased and was 0% for the group that waited more than 6 hours. There were no patients who needed more than 10 hours.

## Discussion

Pieracci and Barie said that the cornerstone of effective treatment for intra-abdominal infection (IAI) with severe sepsis is early and adequate source control [20]. However, there is no definitive answer in the literature to the question of when source control in patients with septic shock should be started. Marshall stated that ‘the immediate priorities in managing the patient with severe sepsis or septic shock are hemodynamic resuscitation. Source control should be instituted as soon as possible after the patient has been stabilized’ [21]. However, sometimes initial resuscitation without infectious source control is not successful; resulting in septic death [2]. It is reasonable to assume that earlier control is better and that the initial resuscitation method is required to improve hemodynamics. Therefore, we developed a protocol that specifies the optimum resuscitation using EGDT and tries to start the surgical procedure immediately even if hemodynamics are still unstable.

Initial resuscitation was performed in accordance with EGDT in all patients. Although the original EGDT protocol requires continuous monitoring of ScvO_2_, the value of ScvO_2_ was monitored intermittently in our protocol. Recently it was shown that intermittent ScvO_2_ monitoring may not be inferior to continuous monitoring during EGDT [22].

The operations were performed using a single methodology for source control and had two points of note. The first was that we performed necrotic intestinal resection and intra-abdominal drainage by laparotomy for all patients. Minimally invasive treatment, such as non-operative management for duodenal ulcer perforation and percutaneous needle drainage for intra-peritoneal abscess, is recommended consistently by Surviving Sepsis Campaign Guidelines (SSCG) [23]. However, in our case, peritonitis in three quadrants or more accounted for 95% of our patients, and we thought that laparotomy was appropriate. The second point of note was that surgical intervention needed an average of 3.4 hours, a relatively long time. In surgery for IAI, we valued the success of the surgical procedure more than reducing the duration of surgery. This is because it has been shown that the outcome of IAI patients who needed re-laparotomy was poorer than that of patients who did not need re-laparotomy [24]. Due to this, we did not need to perform damage control surgery at all. Also, 99% of the patients in this protocol did not require an open abdominal technique owing to intestinal depression by ileus-tube insertion during the laparotomy. We feel that the low frequency of re-laparotomy (11.7%) was a result of this effort.

Patients needed to satisfy the registration criteria for septic shock, and the presence of shock was confirmed by baseline characteristics. Initial resuscitation and surgical procedures were performed using a single methodology according to the protocol. There were no significant differences between survivors and non-survivors in age, gender, perforation site, extent of peritonitis, adequacy of antimicrobial therapy, duration of surgery, or surgical procedure. Under these conditions, statistical examinations clearly proved that time from admission to initiation of surgery for source control is a critical determinant of 60-day survival in patients with GI perforation with associated septic shock. This means that a delay in the initiation of surgery was associated with increased mortality. This finding is a novel, but not unique, therapeutic concept for sepsis. Surgical intervention and antimicrobial therapy target the same source control in the management of sepsis. For non-surgical patients with septic shock, a few clinical studies have revealed that delay of appropriate antimicrobial therapy causes increased mortality [25-27].

It has been shown that delayed surgery in patients with soft-tissue infections increases the risk of mortality [28]. On the other hand, the timing of source control intervention for patients with GI perforation and septic shock has not yet been sufficiently studied [29]. Despite the fact that current international guidelines (SSCG 2012) suggest intervention for source control within the first 12 hours after the diagnosis [23], no definitive clinical studies exist to support this recommendation [29]. This study demonstrated that survival rate decreased as the time to initiation of surgery increased. Also, 60-day survival was 0% when the time to initiation of surgery was greater than 6 hours. We investigated the reasons for these delay of surgical initiation in these patients using the clinical records. We speculate it may be that we needed time to judge the surgical indicators or to secure a surgeon and OR, but we cannot prove it scientifically. Thus, we conclude that we should initiate surgical procedures for infectious source control within a target time of less than 6 hours from admission if the patients have complications from septic shock.

The Infection Diseases Society of America guidelines in 2010 for management of IAI recommend that patients with diffuse peritonitis should undergo emergency surgery as soon as possible, even if ongoing measures to restore physiologic stability need to be continued during the procedure [2]. Recently, De Waele also stated that ‘patients with GI tract perforation and diffuse peritonitis should be operated on within 1–2 hours after diagnosis, irrespective of their response to resuscitation attempts’ [29]. These recommendations are consistent with our findings; however, no clinical data in support of this theory had been published prior to our study.

This study has several limitations. Our examination included a small group of patients and was an observational study performed at a single institution. We consider a randomized controlled trial (RCT) for evaluation of the impact of delay in initiation of surgery to be unethical. However, we think it may be ethically possible to propose an RCT that aims to compare a group with expedited surgical intervention regardless of hemodynamic stabilization and a group with standard surgical initiation after achieving hemodynamic stability following resuscitation. Next, we could not evaluate the impact of EGDT as a preoperative resuscitation method in this protocol. We think that it is necessary to determine a meaning for EGDT by comparing this group of patients with a historical control group of patients before introducing EGDT.

## Conclusion

Within several limitations, we conclude that time from admission to initiation of surgery for source control is a critical determinant of survival in patients with GI perforation with associated septic shock, under the condition that patients were treated with the support of early hemodynamic stabilization by EGDT. The target time for a favorable outcome may be less than 6 hours from admission. To improve the outcome of patients, we should not delay surgical source control procedures assisted by EGDT if patients have the complication of septic shock.

## Key messages

• Shorter time from admission to initiation of surgery for source control was associated with survival in patients with GI perforation with associated septic shock, under the condition that patients were treated with the support of early hemodynamic stabilization by EGDT.

• To improve the outcome of patients, we should not delay surgical source control procedures assisted by EGDT, even if patients have the complication of septic shock. In this analysis, the survival rate was 0% when the time to initiation of surgery was greater than 6 hours.

## Abbreviations

APACHE II: acute physiology and chronic health evaluation II; BGA: blood gas analysis; CT: computed tomography; CVP: central venous pressure; DRPM: Doripenem hydrate; E&CCC: Emergency and Critical Care Center; ED: Emergency Department; EGDT: early goal-directed therapy; EISC: early infectious source control; GI: gastrointestinal; IAI: intra-abdominal infection; MAP: mean arterial pressure; MEPM: Meropenem hydrate; MOD: multiple organ dysfunction score; OR: Operating Room; ScvO2: central venous oxygen saturation; SIRS: systemic inflammatory response syndrome; SOFA: sequential organ failure assessment; TAZ/PIPC: tazobactam/piperacillin; VCM: vancomycin.

## Competing interests

The authors declare they have no competing interests.

## Authors’ contributions

TA as the study director devised the protocol, participated in the design of this study, carried out the data acquisition and analysis, performed the majority of the surgery and participated in drafting the manuscript. KK assisted with study design and participated in drafting the manuscript. YC supervised the statistical analysis and helped to draft the manuscript. DK, TK and AS in the E&CCC treated all patients according to the protocol and participated in collecting the data and critically revised the manuscript for important intellectual content. DK and TK also performed surgeries under the supervision of the study director. KT managed the total coordination of this study and contributed in the draft and editing of the manuscript. All authors read and approved the final manuscript.
